# Glucocorticoids affect bone mineral density and bone remodelling in OVX sheep: A pilot study

**DOI:** 10.1016/j.bonr.2018.11.001

**Published:** 2018-11-15

**Authors:** Diana Cabrera, Frances M. Wolber, Keren Dittmer, Chris Rogers, Anne Ridler, Danielle Aberdein, Tim Parkinson, Paul Chambers, Karl Fraser, Nicole C. Roy, Marlena Kruger

**Affiliations:** a Institute of Veterinary, Animal and Biomedical Sciences, Massey University, Tennent Drive, Palmerston North 4442, New Zealand; b School of Food and Nutrition, Massey University, Tennent Drive, Palmerston North 4442, New Zealand; c Centre for Metabolic Health Research, Massey University, Tennent Drive, Palmerston North 4442, New Zealand; d Riddet Institute, Massey University, Tennent Drive, Palmerston North 4442, New Zealand; e Food Nutrition & Health Team, Food & Bio-Based Products Group, AgResearch Grasslands, Tennent Drive, Palmerston North 4442, New Zealand; f High Nutrition National Science Challenge, Auckland 1142, New Zealand

**Keywords:** Osteoporosis, Sheep, Ovariectomy, Glucocorticoids, Bone mineral density, Bone turnover markers

## Abstract

The aim of this study was to validate the combination of ovariectomy and glucocorticoid treatment in sheep as a large animal model for osteoporosis by measuring the concentration of specific biomarkers in the blood of the sheep and measuring bone loss over five months. Aged Merino ewes were randomly allocated into four groups: control, ovariectomy (OVX), and two OVX groups receiving glucocorticoids—one group once-monthly for five months (OVXG), and the other for two months followed by no treatment for three months (OVXG2). Parameters measured were biochemical markers of bone turnover, areal bone mineral density, volumetric bone mineral density, and total and trabecular bone parameters. Ovariectomy increased the concentrations of bone resorption marker C-terminal telopeptides of type 1 collagen (CTx-1) and bone turnover marker serum osteocalcin (OC) concentrations in the OVX group compared to control sheep. The combination of ovariectomy and glucocorticoid treatment increased the concentrations of CTx-1 and decreased serum OC concentrations in the OVXG group compared to OVXG2. Femur and lumbar spine bone density were lower in experimentally treated groups when compared with the control group. Total and trabecular _v_BMD in the proximal tibia were significantly lower in the treatment groups when compared with the control group. A significant negative correlation between femoral bone density and CTx-1 was found. The results of this study suggest that the combination of OVX and glucocorticoids induces bone loss in a short period of time in sheep.

## Highlights

• Optimise and characterise a sheep model for osteoporosis over five months
• Combining OVX and glucocorticoids induced BMD loss in sheep over five months.
• Bone turnover markers resulted in a reduction of OC and increased CTx-1 markers.

## 1 Introduction

Because of the increased aging population and the rise in the number of bone fractures in elderly people, osteoporosis is considered a major public health issue (Harvey et al., 2010). In women, low levels of oestrogen have effects on bone remodelling, causing loss of bone mass and bone strength due to increased bone resorption and decreased bone deposition (Snyman, 2014). Dual energy x-ray absorptiometry (DXA) and peripheral quantitative computed tomography (pQCT) are useful to measure bone mineral density (BMD) in order to diagnose osteoporosis but cannot predict bone loss (Engelke et al., 2008). To reduce this disease worldwide, assist with understanding the mechanisms involved, and help with early diagnosis, more research must be conducted.

Large animal models are approved by the US Food and Drug Administration for mimicking human osteoporosis in orthopaedic research (Reinwald and Burr, 2008). A well-known method used to induce osteoporosis is ovariectomy (OVX). This method has a direct effect on bone mass due to the induction of oestrogen deficiency. Oestrogen deficiency affects the bone remodelling process, and induces bone loss due to a direct effect on osteoblasts and osteoclasts (Klein-Nulend et al., 2015). Bone formation is inhibited while bone resorption is stimulated, resulting in bone loss (Khosla et al., 2011; Drake et al., 2015). In addition, treatment with glucocorticoids is known to cause secondary osteoporosis, due to increased bone resorption leading to a rapid initial bone loss, and decreased bone formation through suppression of osteoblasts and osteocytes (Compston, 2010; Andreasen et al., 2015).

The OVX sheep is an established large animal model of osteoporosis. Sheep have the advantage that their bone structure and bone metabolism is similar to humans (Newman et al., 1995; Wilke et al., 1997). The model has been used in several studies, and has shown that OVX will induce osteoporosis over six months (Hornby et al., 1995; Turner et al., 1995; Lill et al., 2002; Wu et al., 2008; Zhang et al., 2014; Dias et al., 2018). These studies have reported increased bone turnover in OVX sheep, and all proposed that long periods of time were required for establishing osteoporosis in the sheep (Chavassieux et al., 1993; Chavassieux et al., 1997; MacLeay et al., 2004; Sigrist et al., 2007; Ding et al., 2010). Studies have indicated that when OVX is combined with glucocorticoids, further bone loss is induced (Lill et al., 2002; Schorlemmer et al., 2005; Ding et al., 2010; Veigel et al., 2011). Therefore, a combination of OVX with glucocorticoids will increase bone loss in sheep; using both OVX and glucocorticoids could reduce the time needed to develop osteoporosis, and the trial time could be reduced. However, weekly treatment with glucocorticoids after long-term treatment has been shown to negatively impact the animals' health with severe side effects such as infections and hair loss (Lill et al., 2002; Schorlemmer et al., 2003). More importantly, how glucocorticoid withdrawal effects the rebound of bone is not yet fully understood in OVX sheep. Thus, strategies to reduce future complications of long-term glucocorticoid treatment in OVX sheep need to be considered.

It was hypothesised that ovariectomising sheep in combination with monthly injections of glucocorticoids would result in decreased BMD and increased plasma bone remodelling marker concentration over a shorter period of time than previously reported, while eliminating the negative side effects. The aims of this study were to: 1) validate the OVX sheep model by measuring the concentration of serum biomarkers and bone loss over a five-month period; and 2) investigate the effects of short- and long-term glucocorticoid administration on BMD in the lumbar spine, femur and tibia, as well as bone biomarkers, in the OVX sheep.

## 2 Materials and methods

### 2.1 Experimental animals

**Fig. 1:**
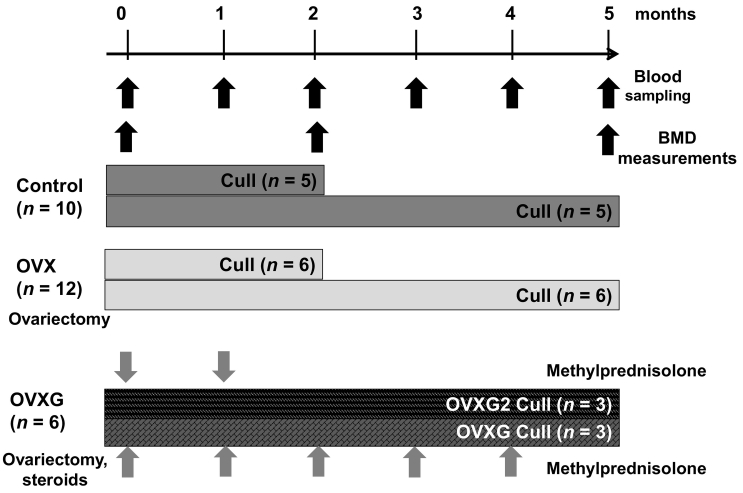
Flow diagram of the experimental study. Merino ewes were randomly divided into groups, 10 underwent no treatment (control group), 12 ewes underwent ovariectomy (OVX), and the remaining 6 ewes underwent OVX and received an injectable suspension of 400 mg methylprednisolone (OVXG group). Half of the ewes in the OVXG group received the methylprednisolone treatment for five months, while the other half received this treatment for only two months (OVXG2 group). Blood samples were collected monthly. BMD measurements were done at baseline (month zero) by pQCT, and at post-cull (month two or month five) by pQCT and DXA. Half of the sheep in the control group and OVX group were euthanised after two months, and the remaining sheep for each group were euthanised after five months. All the sheep in the OVXG group and OVXG2 were euthanised after five months.

Aged Merino ewes (7–9 years old, *n* = 28) were obtained from a commercial farm in the Whanganui region, New Zealand. The animals were housed in a barn and adapted to indoor pens with rubber mat flooring. Sheep had free access to water and were fed the control diet described below for 20 days. After adaptation, sheep were randomly allocated into groups: control group (*n* = 10), the OVX group (*n* = 12), and two OVX groups receiving glucocorticoids, one group once a month for five months (OVXG) (*n* = 3) and the other group once a month for two months followed by no treatment for three months (OVXG2) (*n* = 3) (Fig. 1).

Control and low-calcium sheep pellet concentrate diets were formulated from soya, brollard and wheat to contain 11% protein, 34% starch, and 29% nondigestible fibre, as well as >2000 IU/kg vitamin D3/cholecalciferol and <200 IU/kg vitamin D2 (ergocalciferol), which are required for calcium absorption. While 2–4 g/kg calcium is sufficient for sheep under normal circumstances, the animals in the current study were housed indoors and had limited exposure to sunlight and no exposure to sun-cured forage. Under these circumstances, calcium absorption was likely to be impaired. It was considered undesirable to risk vitamin D toxicity by increasing the vitamin D levels to aid calcium absorption in the gut, so the dietary calcium levels were increased to compensate. Ewes were fed at approximately 200 g/sheep/day. Wheat straw and water were available to all sheep *ad libitum* throughout the study.

Sheep were anaesthetised using 0.5 mg/kg diazepam (Ilium, Troy Laboratories Pty Ltd., Glendenning, Australia) and 10 mg/kg ketamine (Phoenix Pharm Distributors Ltd., Auckland, New Zealand) intravenously, followed by endotracheal intubation and maintenance of anaesthesia with halothane. The distal tibia from each sheep was pQCT-scanned under general anaesthesia. The sheep, except for the control group, underwent bilateral OVX. The OVX was performed *via* a ventral abdominal incision. The linea alba and subcutaneous tissues were closed using 1 USP polydioxanone (PDS II, Ethicon Inc., USA) and the skin with 1 USP nylon suture (Kruuse A/S, Langeskov, Denmark), and the sutures were removed 14 d after the surgeries. Prior to extubation, each sheep was given 20 mg/kg meloxicam (Metacam, Boehringer Ingelheim GmbH, Ingelheim am Rhein, Germany) for pain. At the same time, the 10 sheep in the control group were anaesthetised but no surgery was performed. Post-surgery, until suture removal, sheep were examined twice daily. After suture removal, sheep were checked twice daily and food, water, activity and hair condition monitored.

On day two after OVX, six OVX sheep were administered an injectable suspension of 400 mg methylprednisolone (Vetacortyl®_,_ Vetoquinol SA, Lure Cedex, France) subcutaneously in the neck. Methylprednisolone treatment started before the sutures were removed. Three of the sheep were given four further treatments at the same dose once a month (OVXG). The remaining three sheep received the initial treatment and then an additional treatment was given one month later, after which the treatment was discontinued in order to observe the effect on bone after glucocorticoid withdrawal (OVXG2).

All experimental procedures were approved by the Massey University Animal Ethics committee (approval number 14/103) and performed according to the Code of Ethical Conduct for the use of live animals for research at Massey University, Palmerston North, New Zealand.

### 2.2 Blood sampling

A blood sample was collected from all sheep *via* jugular venepuncture into 10 mL plain (serum), heparin and EDTA tubes (BD Vacutainer, Franklin Lakes, New Jersey, USA) on the day of surgery (month 0) and then monthly for the duration of the study until the animals were euthanised. Blood samples were taken between 08:00 and 10:00 h to limit any circadian effect.

The EDTA and heparin blood tubes were centrifuged at 2000*g* for 10 min and the plasma removed and stored at −80 °C for later analysis. The tubes with no anticoagulant were left at room temperature for 45 min to clot prior to being processed as above.

### 2.3 Euthanasia and dissection

Half the ewes from the control group (*n* = 5) and half from the OVX group (*n* = 6) were euthanised using a captive bolt followed by exsanguination at two months. At the end of the study (five months), the remaining ewes from the control group (*n* = 5) and the OVX group (*n* = 6), and all of the ewes from the OVXG (*n* = 3) and the OVXG2 (*n* = 3) groups, were similarly euthanised. After euthanasia, the femurs, lumbar spines (LSs) and tibias were collected and stored at 4 °C. Samples of ileum, liver, kidney, heart and lung were collected and stored in 10% neutral buffered formalin until processing for histology. Microscopic examination of 3 μm paraffin-embedded haematoxylin- and eosin-stained sections confirmed the absence of unrelated disease.

### 2.4 Analysis of biochemical markers of bone

Osteocalcin (OC) was measured in serum. Samples were tested in duplicate with an immunoassay kit (MicroVue Osteocalcin, San Diego, CA, USA) which uses a monoclonal mouse antibody that recognises only intact (*de novo*) OC in serum. The MicroVue immunoassay used has cross-reactivity with sheep species (Dias et al., 2008).

Serum C-terminal cross-linked telopeptides of type I collagen (CTx-1) were measured in heparinised plasma. Samples were tested in duplicate using the Serum CrossLaps ELISA kit (IDS, London, UK), which uses two highly specific monoclonal antibodies. The IDS immunoassay has been used for sheep (Wilkens et al., 2014).

EL_x_ 808 ultramicroplate reader (Bio-tek Instruments Inc., USA) was used to generate a standard curve from each ELISA kit and measure samples. Then, sample absolute concentrations were extrapolated from the standard curve. OC and CTx-1 were measured in the sheep at baseline (month zero), two months and five months of the trial.

### 2.5 Analysis of bone parameters

#### 2.5.1 Dual-energy x-ray absorptiometry (DXA)

Lumbar spine and femur were thawed at room temperature. *Ex vivo* measurements of bone mineral component (BMC) and areal BMD (aBMD) for the lumbar spine and femur were determined using the Hologic Discovery A Bone Densitometer (Hologic Discovery **QDR 4500A** densitometer, Hologic Inc., Bedford, Massachusetts, USA).

#### 2.5.2 Peripheral quantitative computed tomography (pQCT)

Three-dimensional analyses of the left tibial bone were performed using pQCT. The first initial baseline scan was obtained after the sheep were anaesthetised for surgery. *In vivo* measurements were made of total volumetric BMD (_v_BMD), bone area, bone mineral content (BMC), cortical/subcortical and trabecular _v_BMD, cortical/subcortical and trabecular bone area, cortical/subcortical and trabecular BMC, and geometric variables of the mid-metaphysis 10 mm from the distal surface of bone and at the mid-diaphysis 70 mm from the distal end.

Left tibias were thawed at room temperature. *Ex vivo* measurements were made of total *v*BMD, bone area, BMC, cortical/subcortical and trabecular _v_BMD, cortical/subcortical and trabecular bone area, cortical/subcortical and trabecular BMC and geometric variables of the proximal tibia, the mid-metaphysis and at the mid-diaphysis using the XCT2000 peripheral quantitative computed tomography (pQCT) scanner (Stratec Medizintechnik GmbH, Pforzheim, Germany). The measurement site was located at the proximal metaphysis 15 mm from the proximal surface of bone. The pQCT scan for the *in vivo* and *ex vivo* measurements had a voxel size of 0.3 mm.

Norland/Stratec XCT 5.50 software was used for scan imaging analysis. The trabecular compartment was obtained at the central 45% of bone at the trabecular sites. The manufacturer's contour threshold was set at 280 mg/cm^3^ to separate soft tissue from the outer edge of bone. For cortical bone, the threshold was set at 710 mg/cm^3^.

### 2.6 Statistical analyses

Data for bone marker concentrations were analysed to report the effects of treatments at specified time points. For analysis of percentage of change from baseline, baseline results were included as covariate and the analysis was based on the results from month two and month five. Data for lumbar spine, femoral and tibial parameters were analysed to test the effect of the OVX plus glucocorticoid treatments after sacrifice at month two and month five. Data are expressed as mean and standard deviation (SD). Statistical analyses were performed with the nonparametric Kruskal–Wallis test to assess whether the treatment means were significantly different. The nonparametric Kruskal–Wallis test was followed by post-hoc comparisons of treatment means using Dunn's post-hoc test. Statistical analyses on data were conducted using R (R 3.1.1, R Foundation for Statistical Computing, Vienna, Austria). We performed the Spearman rank-order correlation to assess the association between BMDs of the lumbar spine, femur and tibia with bone biomarker concentrations. This test was done using Minitab 17.2.1 (Minitab Inc., Pennsylvania, USA). In all statistical analyses, *p*-values <0.05 were considered statistically significant.

## 3 Results

### 3.1 Effect of OVX alone or combined with glucocorticoid on biochemical markers of bone in the sheep model

During bone loss in osteoporosis, measurable metabolites are released in the blood, including OC and CTx-1. OC is a noncollagenous bone protein, which is released from osteoblasts during bone formation and from bone matrix during bone resorption. CTx-1 is a collagen breakdown product, which is released during bone resorption (Swaminathan, 2001; Allen, 2003; Garnero et al., 2003).

**Fig. 2:**
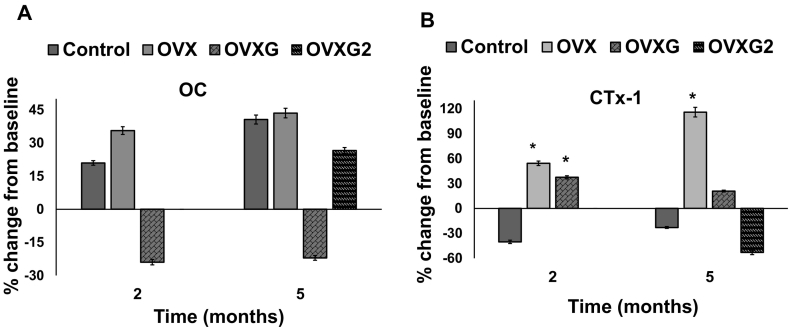
The concentrations of OC and CTx-1 in the serum of the OVX sheep. Percentage change in the bone turnover marker serum OC (A) and percentage change in the bone resorption marker CTx-1 (B) compared with the pretreatment baseline from Merino ewes that were either ovariectomised (OVX), ovariectomised and given five doses of glucocorticoid at monthly intervals (OVXG), ovariectomised and given two doses glucocorticoid at monthly intervals (OVXG2), or received no treatments (Control). OC and CTx-1 were measured at two months and five months after initiation of the treatments. The results are presented as serum OC and CTx-1 mean percent change from baseline. *p-value <0.05. Error bars denote standard error of mean.

Fig. 2 and Table 1 show the changes in OC and CTx-1 markers over five months of treatment in OVX sheep. The percentage change from baseline of individual sheep is shown for each parameter (Fig. 2). Sheep exhibited serum OC concentration mean values between 12 and 15 ng/mL at baseline (Table 1). Serum OC concentration in the control sheep increased by 22% at two months, and increased further by 40% at five months (Fig. 2A). Similarly, serum OC increased by 35% (*p*-value = 0.337) within two months and by 43% (*p*-value = 0.384) at five months compared to baseline in OVX sheep. On the other hand, serum OC concentration decreased by 24% (*p*-value = 0.040) at two months and remained at this decreased level (*p*-value = 0.099) at five months compared to baseline in the OVXG group. However, when the glucocorticoids were discontinued in the OVXG2 group at two months, the serum OC concentrations observed at five months increased by 26% (*p*-value = 0.209), compared to baseline in the OVXG2 group.

**Table 1:** Changes of OC and CTx-1 over five months in the OVX sheep.

Time (Month)	OC (ng/mL)					CTx-1 (ng/mL)				
	Control	OVX	OVXG		*p*-value	Control	OVX	OVXG		*p*-value
0	12.19 (4.97)	15.07 (4.86)	15.86 (6.66)		0.107	0.96 (0.78)	0.71 (0.36)	0.74 (0.18)		0.87
2	13.17 (4.17)	19.33 (6.72)	10.78 (7.48)		0.013	0.61 (1.13)	0.93 (0.56)	0.87 (0.36)		0.005
5	16.25 (7.24)	18.38 (8.87)	12.71^a^ (4.13)	16.95^b^ (4.33)	0.77	0.28 (0.04)	0.95 (0.58)	0.66^a^ (0.14)	0.4^b^ (0.24)	0.01

Data are presented as serum OC and CTx-1 mean (SD). The resulting *p*-values obtained by Kruskal–Wallis are given. *p*-value <0.05 was considered as statistically significant.

a Half of the ewes in the OVXG group received the methylprednisolone treatment for five months (OVXG group).

b Half of the ewes in the OVXG group received the methylprednisolone treatment for only two months (OVXG2 group).

The experimental treatment had a significant effect on the bone resorption marker, serum CTx-1 concentration (Fig. 2B; Table 1). Sheep exhibited serum CTx-1 concentration mean values between 0.7 and 0.9 (ng/mL) at baseline. Serum CTx-1 concentration in the control sheep decreased by 40% at two months and again by 23% at five months. In contrast, serum CTx-1 concentration in the OVX sheep increased within two months by 54% (*p*-value = 0.002) from baseline, increasing to 115% (*p*-value = 0.005) at five months compared to baseline. Further, serum CTx-1 concentrations increased 37% (*p*-value = 0.045) from baseline within two months of glucocorticoid treatment in the OVXG group. Surprisingly, these concentrations decreased by 20% (*p*-value = 0.116) after five months of glucocorticoid treatment when compared to baseline. Discontinuing glucocorticoids at two months decreased serum CTx-1 concentrations by 52% (*p*-value = 0.112) at five months from baseline in the OVXG2 group.

### 3.2 Effect of OVX alone or combined with glucocorticoid on bone parameters in the sheep model

**Fig. 3:**
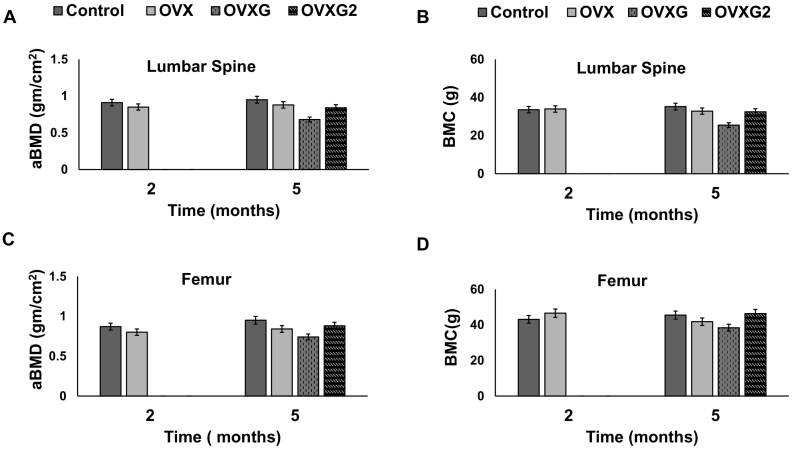
Bone parameters of lumbar spine and femur at month two and month five measured by DXA. (A) lumbar spine aBMD, (B) lumbar spine BMC, (C) femoral aBMD and (D) femoral BMC are shown for half of the ewes euthanised at month two in the control group and OVX group, and for the remaining ewes at month five for each group. Data is presented as mean and 95% confidence intervals.

The results of DXA scans of the lumbar spine and femur are shown in Fig. 3. After OVX, the lumbar spine and femur parameters did not differ between treatments compared with controls at two months (Fig. 3). Lumbar spine aBMD was slightly reduced by 6% (*p*-value = 0.36) after OVX, but lumbar spine BMC was not affected two months after OVX. In addition, femoral aBMD did not vary between the control group and OVX group at two months.

Similarly, the lumbar spine and femur did not show any significant differences in bone parameters compared with controls at five months. However, the lumbar spine and femoral aBMD of experimentally treated animals were decreased from controls at five months (Fig. 3). The highest impact on aBMD was found in the OVXG group. Lumbar spine aBMD was reduced by 7%, 28% and 12% (*p*-value = 0.06) in the OVX, OVXG and OVXG2 groups, respectively (Fig. 3A). Lumbar spine BMC was decreased by 6.7%, 27% and 7% (*p*-value = 0.15) in the OVX, OVXG and OVXG2 groups, respectively (Fig. 3B). Femoral aBMD was reduced by 10%, 21% and 6% (*p*-value = 0.06) in the OVX, OVXG and OVXG2 groups, respectively (Fig. 3C). Femoral BMC was slightly but not significantly reduced after five months of treatment in all treated groups (*p*-value = 0.19) (Fig. 3D).

**Fig. 4:**
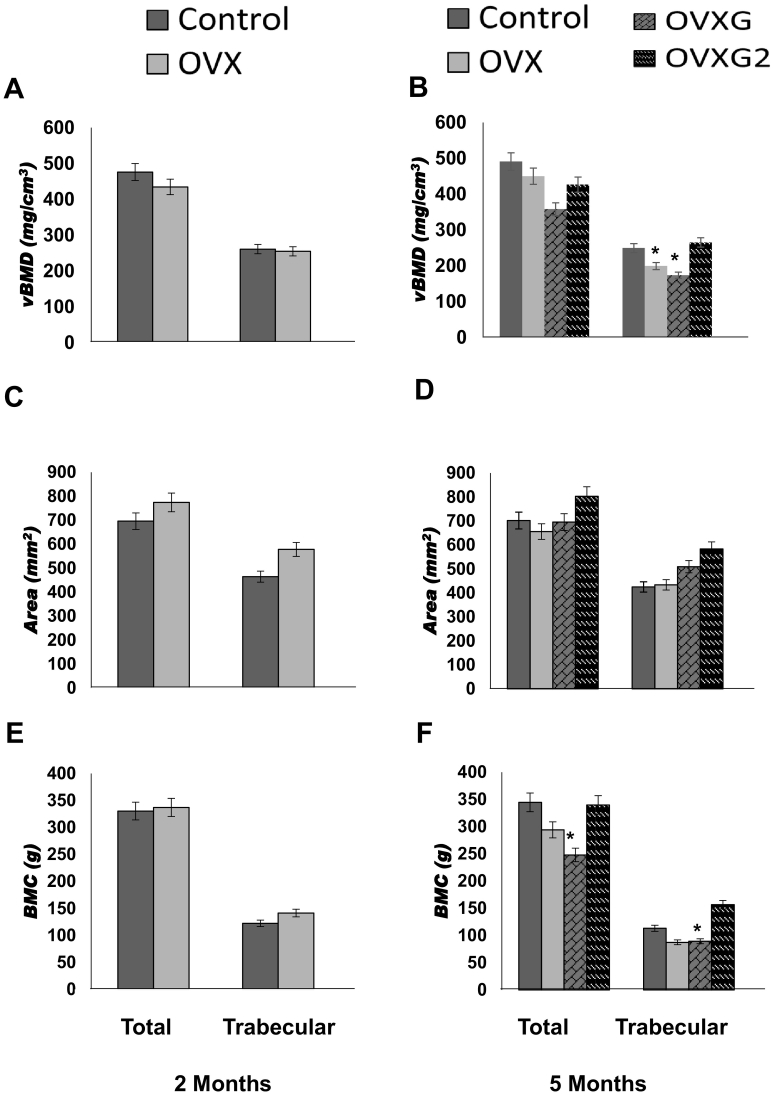
Bone variables at the proximal metaphysis of the tibia at month two and month five measured by pQCT. Total and trabecular tibial vBMD, area and BMC (A), (C) and (E), respectively, at month two. Total and trabecular tibial vBMD, area and BMC (B), (D) and (F), respectively, at month five are shown for half of the ewes euthanised at month two in the control group and OVX group; and the remaining ewes at month five for each group. Data is presented as mean and 95% confidence intervals. *p-value <0.05 was considered as statistically significant.

The results of pQCT scans of the proximal metaphysis of the tibia post-cull are shown in Fig. 4. The proximal metaphysis of the tibia at two months (Fig. 4A, C, E) did not show significant differences between control and OVX treatment groups (*p*-value >0.05). Total vBMD was reduced by 8% after two months of treatment (*p*-value = 0.1). Bone area and BMC did not vary between groups at two months. No difference could be found in trabecular vBMD (*p*-value = 0.72), trabecular area (*p*-value = 0.14) and trabecular BMC (*p*-value = 0.58).

The proximal metaphysis of the tibia at five months (Fig. 4B, D, F) showed a significant treatment effect. Tibial vBMD was decreased by 8% (*p*-value = 0.052), 27% (*p*-value = 0.0009) and 13% (*p*-value = 0.057) in the OVX, OVXG and OVXG2, respectively (Fig. 4B). BMC was decreased by 14% (*p*-value 0.051), 28% (*p*-value = 0.001) and 1% (*p*-value = 0.293) in the OVX, OVXG and OVXG2, respectively (Fig. 4F). Trabecular vBMD was reduced by 20% (*p*-value = 0.019) and 30% (*p*-value = 0.003) in the OVX and OVXG, respectively (Fig. 4B). Trabecular BMC was reduced by 22% (*p*-value = 0.044) and 20% (*p*-value = 0.109) in the OVX and OVXG, respectively (Fig. 4F).

The data were pooled from all the treatment groups at two months and five months, and further analyses performed to investigate any possible associations between parameters. Relationships of BMD of femur and tibia with OC and CTx-1 concentrations in sheep at two months were not significant using Spearman's rank-order correlations (Table 2). However, a significant correlation was observed between lumbar spine aBMD and OC (*r* = −0.636; *p*-value = 0.048).

**Table 2:** Correlation between biochemical markers and BMD of lumbar spine, femur and tibia in OVX sheep at month two and month five of the study.

	OC		CTx-1	
	Spearman's correlation	*p*-value	Spearman's correlation	*p*-value
Month 2				
Lumbar spine aBMD	−0.636	0.048	−0.345	0.298
Femur aBMD	−0.261	0.466	0.346	0.297

Tibia				
vBMD	−0.418	0.229	−0.336	0.312
Trabecular vBMD	−0.479	0.162	−0.409	0.212

Month 5				
Lumbar spine aBMD	0.262	0.309	−0.212	0.414
Femur aBMD	0.140	0.593	−0.505	0.039

Tibia				
vBMD	0.130	0.619	−0.284	0.269
Trabecular vBMD	0.184	0.480	−0.404	0.107

*p*-value <0.05 was considered as statistically significant.

The correlations between biochemical markers and BMD of lumbar spine, femur and tibia at five months were positive for OC and negative for CTx-1, but not significant using Spearman's rank-order correlation (Table 2). However, a strong and significant negative correlation between femoral aBMD and CTx-1 was found (*r* = −0.505; *p*-value = 0.039).

## 4 Discussion

In this study, we applied a combination of advanced age, OVX and glucocorticoid treatment to determine bone mass quantity, quality and bone markers using sheep as a large animal model for postmenopausal osteoporosis. A period of five months caused a decreased BMD in the glucocorticoid OVX sheep with a significant reduction of aBMD in the lumbar spine and femur as measured by DXA, and a significant decrease in tibial cortical and trabecular vBMD as measured by pQCT. The decreases in aBMD and vBMD were greater in the glucocorticoid-treated ovariectomised sheep compared with the control groups and those that only underwent OVX. Interestingly, after cessation of glucocorticoid administration, there was a recovery of bone mass in sheep treated with glucocorticoids for only two months. Serum biomarkers were increased in the ovariectomised sheep; however, those same serum markers were reduced in the glucocorticoid-treated OVX sheep.

### 4.1 Effect of OVX on bone biomarkers and bone parameters in sheep

Bone turnover markers can be used as measures of the status of bone remodelling. The bone remodelling depends on the synchronization of bone resorption cells—osteoclasts and subsequent bone formation cells—osteoblasts. During menopause, bone resorption increases as a result of oestrogen deficiency as well as bone formation to fill the bone cavities that have been destroyed (Vasikaran et al., 2011). In this study, after five months of OVX on a diet providing the minimum required level of calcium, the bone resorption (CTx-1) serum biomarker increased at two months in the OVX group and remained increased until the end of the study. Similarly, serum OC increased after OVX and remained increased during the study. These results are in agreement with Kiełbowicz et al. (2015) that reported increased serum CTx-1 concentrations in OVX sheep. Other authors have reported an increase in urinary CTx-1 three months, and serum pyridinoline concentrations two months, after OVX (Chavassieux et al., 2001; Sigrist et al., 2007). Other studies also support these findings with regard to serum OC concentration; also showing that serum OC concentrations increased after OVX treatment in sheep (Chavassieux et al., 2001; Johnson et al., 2002; Newton et al., 2004). It was also observed that OC and CTx-1 concentrations were significantly negatively correlated with lumbar spine aBMD and femoral aBMD, respectively. These results suggest a possible association between bone turnover and bone density, where bone turnover is able to predict a reduction of BMD. These increased serum concentrations of OC and CTx-1 reflect increased bone resorption in lumbar spine and femur due to OVX promoting bone loss and increasing bone turnover (Yoon et al., 2012). These findings suggest that a two-month time point may be sufficient for assessing the early effects of OVX on bone resorption and can reliably predict changes in bone structure.

In this study, a reduction of lumbar and femur BMD was found, as assessed by DXA, when compared with the control group that was not ovariectomised and was fed a diet with plentiful calcium. These results are similar to those reported by previous studies; with a decrease of aBMD reported in the spine at six months (Turner et al., 1995) and 12 months after OVX (Hornby et al., 1995; Wu et al., 2008; Zhang et al., 2014).

In this study, tibia vBMD, trabecular vBMD and cortical/subcortical vBMD as assessed by pQCT showed a decrease in bone mass after five months of OVX. However, published reports are controversial, as two of the few studies published to date reported no significant differences in trabecular vBMD in the proximal tibia after twelve months of OVX in sheep (Augat et al., 2003). However, Sigrist et al. (2007) observed a significant decrease in trabecular vBMD of the distal radius after four months of OVX. Taken together, these findings suggest that five months or less is a sufficient time period post-ovariectomy to reliably measure physical changes in BMD in the sheep model, where DXA and pQCT are suitable methods of measurement to assess BMD.

### 4.2 Effect of glucocorticoid treatment on bone biomarkers and bone parameters in OVX sheep

The combination of OVX, minimal dietary calcium, and glucocorticoid treatment caused a transient increase in the serum CTx-1 concentrations at two months, after which concentrations were lowered by the end of the study. This result is supported by previous studies, where serum CTx-1 concentrations increased during the first months in OVXG sheep but decreased thereafter (Zarrinkalam et al., 2009; Ding et al., 2010; Andreasen et al., 2015). The bone resorption marker CTx-1 has been shown to increase during the administration of steroids. The direct effect of glucocorticoids may be in promoting osteoclastogenesis (Takuma et al., 2003) and having an anti-apoptotic effect on osteoclasts (Jia et al., 2006).

In contrast, the serum OC concentration of sheep treated with glucocorticoids for five months decreased until the end of this study. This result is similar to some previous studies, where serum OC concentration rapidly decreased (Chavassieux et al., 1993; Chavassieux et al., 1997; Ding et al., 2010) and remained suppressed in sheep (Andreasen et al., 2015) with the combination of OVX and glucocorticoid treatment. However, it is well known that glucocorticoids affect osteoblasts and osteocytes, decreasing their lifespan and bone formation, as reflected by the reduction of serum OC concentrations (Van Staa et al., 2002).

An unexpected outcome of the current study is that the serum OC and CTx-1 concentrations in the group that received only two months of glucocorticoids were closer to the control group after cessation of glucocorticoid administration. This finding is supported by Ding et al. (2010), where serum OC recovered after the glucocorticoid treatment was discontinued at seven months in their study.

In the present study, BMD decreased after five months of the combination of OVX and glucocorticoids in the lumbar spine, femur and tibia, although this reduction varied according to the skeletal site. Previous research reported decreased cortical and trabecular vBMD in the lumbar spine and distal radius with OVX plus glucocorticoids in sheep over a period of six months (Lill et al., 2002), tibia (Schorlemmer et al., 2005) at 12 months and iliac bone (Andreasen et al., 2015) over a period of seven months. Moreover, other studies reported the reduction of cortical and trabecular vBMD in the lumbar spine, distal radius and proximal tibia with OVX, glucocorticoids and diet in sheep (Lill et al., 2002; Augat et al., 2003; Veigel et al., 2011). In this study, a significant reduction of trabecular compartment was detected during the administration of glucocorticoids, and this contributed to lower vBMD, which was observed after five months of treatment. Thus, the combination of OVX and glucocorticoids accelerates a profound bone loss in a short time, by inducing bone resorption and suppressing bone formation.

Sheep is recognised as a large animal model for studying human osteoporosis. The OVX sheep model is useful to understand the mechanisms that contribute to bone loss. However, there has been controversy about the optimal period of time for achieving bone loss due to there is no agreement within the literature of the time necessary to establish an osteoporotic (osteopenic) bone in OVX sheep. There have been several hypotheses proposed on the time required to induce measurable bone loss in sheep (Chavassieux et al., 2001; Les et al., 2005; Sigrist et al., 2007), although the duration required remains controversial due to the different breeds used in each study, the small number of animals per sampling, and the different approaches used to evaluate bone quantity and quality (Lill et al., 2002; Ding et al., 2010; Veigel et al., 2011). However, in this study, it was found that a short-term ovariectomised glucocorticoid-treated model in Merino ewes can be used to induce bone loss without the side effects and complications of using glucocorticoids, such as hair loss, infections, and particularly, ethical concerns around animal welfare for the use of research animals as experimental units (Egermann et al., 2008). These results showed that five months was sufficient to induce bone loss using a combination of OVX and glucocorticoid treatment in Merino ewes, and that once-monthly injections of glucocorticoids resulted in no clinical infections or negative dermatological effects.

The limitations of this study include the small sample size and that bone mineral density was determined as a function of treatment evaluated in OVX sheep. Future studies such as mechanical properties of bone and changes in its extracellular matrix components are required to fully investigate bone quality in OVX sheep. However, the strengths are the induction of bone loss in a short period of time and the assessment of bone mass during and after the cessation of glucocorticoids combined with OVX. Therefore, this present project should be considered a pilot study, where a combination of OVX and glucocorticoids was used to induce bone loss over a short period of time in sheep. A reduction in time may allow the sheep model to be used more frequently, to test novel therapies for postmenopausal women.

## 5 Conclusions

This pilot study suggested that the interaction of OVX and glucocorticoids plays a significant role and induces bone loss in sheep over five months, and this bone loss is similar to that seen in humans. The combination of OVX and length of glucocorticoid treatment should be carefully considered when designing studies using OVX sheep. Further, the use of experimental animal models for studying bone loss to test new experimental drug therapies and orthopaedic implants offers opportunities to enhance our understanding of bone diseases, particularly postmenopausal osteoporosis.

## Transparency document

Transparency document. Image 1

## Conflict of interest

The authors have no conflict of interest.
